# Biocementation of Pyrite Tailings Using Microbially Induced Calcite Carbonate Precipitation

**DOI:** 10.3390/molecules27113608

**Published:** 2022-06-04

**Authors:** Bo Kang, Fusheng Zha, Weihao Deng, Runkai Wang, Xianguo Sun, Zhitang Lu

**Affiliations:** 1 School of Resource and Environmental Engineering, Hefei University of Technology, Hefei 230009, China; kangbo@hfut.edu.cn (B.K.); 12132195@mail.sustech.edu.cn (W.D.); runkai_king@163.com (R.W.); zhitang_lu@163.com (Z.L.); 2 State Environmental Protection Key Laboratory of Integrated Surface Water-Groundwater Pollution Control, School of Environmental Science and Engineering, Southern University of Science and Technology, Shenzhen 518055, China; 3 Anhui Huizi Construction Engineering Co., Ltd., Wuhu 241004, China; ss8211050302@163.com

**Keywords:** acid mine drainage (AMD), microbially induced calcium carbonate precipitation (MICP), source control, biochemical and physicochemical reactions

## Abstract

Tailing sand contains a large number of heavy metals and sulfides that are prone to forming acid mine drainage (AMD), which pollutes the surrounding surface environment and groundwater resources and damages the ecological environment. Microbially induced calcium carbonate precipitation (MICP) technology can biocement heavy metals and sulfides in tailing sand and prevent pollution via source control. In this study, through an unconfined compressive strength test, permeability test, and toxic leaching test (TCLP), the curing effect of MICP was investigated in the laboratory and the effect of grouting rounds on curing was also analyzed. In addition, the curing mechanism of MICP was studied by means of Fourier transform infrared spectroscopy (FTIR), thermogravimetric analysis (TGA), X-ray diffraction spectroscopy (XRD), and scanning electron microscopy (SEM). The experimental results showed that MICP could induce calcium carbonate precipitation through relatively complex biochemical and physicochemical reactions to achieve the immobilization of heavy metals and sulfides and significantly reduce the impact of tailing sand on the surrounding environment.

## 1. Introduction

One of the most relevant environmental concerns currently faced by the mining industry is acid mine drainage (AMD) [1]. The sulfur minerals contained in pyrite tailings undergo an oxidation reaction under the joint action of chemical oxidants and *Thiobacillus ferro* oxidants to form AMD [2]. The pH value of AMD is usually very low (in severe cases, the pH value can reach 2). In addition, it is rich in SO_4_^2−^ ions, Fe^3+^ ions, and easy to leach toxic elements such as lead, arsenic, chromium, cadmium, and copper from waste ore [3]. AMD has become a major obstacle to sustainable development because of its large volume, wide range, serious pollution, and difficult governance [4]. AMD source control technology can inhibit the oxidation of sulfur minerals through some technical means according to the formation mechanism of acid wastewater from tailings, so as to control the formation of acid water. The oxidation of pyrite is a slow process if it is covered with water or other inhibiting layers. Therefore, some researchers have proposed the use of physical barriers to inhibit the oxidation responsible for AMD, e.g., fly ash and sludge layers [5,6] or natural soil [7].

Microbially induced carbonate precipitation (MICP) technology has several advantages: it is a low-carbon, high-efficiency, green, and ecological method [8,9,10]. It can generate carbonate precipitate by combining CO_3_^2−^ produced during microbial metabolism with Ca^2+^ ions to enhance soil strength, reduce soil permeability, and immobilize heavy metals [11,12,13,14,15]. MICP technology has been proven to be effective in improving soil strength [16,17,18,19,20,21] and cementing heavy metals [22,23]. Many recent studies have shown that MICP technology can effectively reduce the porosity and permeability of porous media. For example, Stabnikov et al. [24] reduced the permeability of sand columns by three orders of magnitude through the process of biomineralization. Gustavo et al. [25] used two ureolytic strains (UB3 and UB5) of *Sporosarcina luteola* investigated to induce the sequestration of metals by the precipitation of carbonates in vitro and under microcosm conditions. Zeinab Piervandi et al. [26] used indigenous bacteria to fix heavy metal elements in tailings sand and showed that the passivation layer could adsorb heavy metals. Heavy metals could also be co-precipitated with the passivation layer, which brought about the main methods used for removing toxic elements. The practice has demonstrated that MICP technology can reduce the acid mine wastewater pollution of groundwater and surface by forming a biocement barrier with good durability on the surface of the tailings sand accumulation [27]. In addition, the resulting calcium carbonate can also co-precipitate with heavy metal ions in the tailings sand, preventing the heavy metals from diffusing into the surrounding environment [28,29,30,31].

In this study, the effects of different grouting times on the engineering properties and environmental effects of MICP-biocemented pyrite tailings were explored under the following optimal conditions [32]: (1) the bacterial liquid had an OD_600_ value of 1.8; (2) the concentration of cementitious liquid was 1.0 mol/L; and (3) the urea-to-calcium-chloride ratio was 1:1. The mineral composition, thermal stability, and microstructure of the biocemented samples were studied via X-ray diffraction (XRD), Fourier transform infrared absorption spectroscopy (FTIR), thermogravimetric analysis (TGA), and scanning electron microscopy (SEM). The biochemical mechanism underpinning the biocementation of pyrite tailings by MICP was revealed through macroscopic mechanical properties and microstructure characteristics, which indicated that the technique may have a significant impact on environmental remediation.

## 2. Materials and Methods

### 2.1. Pyrite Tailings 

**Figure 1:**
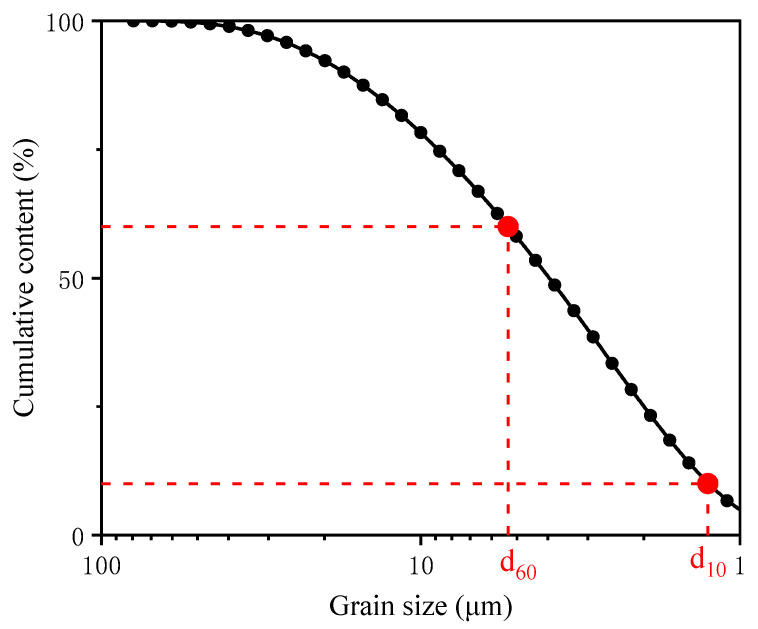
The gradation curve of the sample tailings.

The pyrite tailings sand used in this study was collected from Xinqiao tailings pond, Tongling City, Anhui Province. The pyrite tailings were in a relatively wet state, and the pH value of the surface pyrite tailings was measured as 7.02. The loose bulk density of the tailings sand was determined using the ring knife method, and the specific gravity of the tailings was measured using the pycnometer method (JTGE40-2007); the recorded values were 1.33 g/cm^3^ and 3.02, respectively. The test results are shown in Figure 1. It can be observed that d_60_ was 5.32 μm, and d_10_ was 1.26 μm. Therefore, we calculated the uniformity coefficient (Cu) as 1.22, which is smaller than 5. These results indicated that most of the tailings sand particles were well-graded.

The XRF results of the pyrite tailings are shown in Table 1. The pH of the pyrite tailings sand was slightly acidic. The pH value of pyrite tailings sand that was left for a period of time was 4.75, indicating that the sulfur content was high, which was consistent with the XRF test results. 

In order to clarify the content and migration ability of heavy metals in the sample, the TCLP method was used; in addition, the SO_4_^2−^ content was detected via ion chromatography. The detection results are shown in Table 2.

### 2.2. Microorganisms and Culture Medium

*Sporosarcina pasteurii* (*S. pasteurii*, ATCC 6452), a non-pathogenic bacterium with high urease activity, was used for MICP in this study [33,34]. The bacterial solution was prepared by inoculating bacterial colonies into the NH_4_-YE solution medium (20 g/L yeast extract, 10 g/L (NH_4_)_2_SO_4_, and 0.13 M Tris-base) and was then subjected to shaking incubation at 30 °C for approximately 24 h until the optical density at 600 nm (OD_600_) reached the designated value. The OD_600_ value was measured using a UV–Visible spectrometer (Shanghai Analytical Instruments Co., Ltd., Shanghai, China). The prepared bacterial solution was then stored at 4 °C. Prior to its use in the experiment, the solution was firstly re-harvested in a fresh growth medium. When bacterial cells reached the exponential growth stage during resuscitation, they were inoculated into the solution for the formal test. The NH_4_-YE medium, CaCl_2_, and urea solutions were sterilized by autoclaving at 121 °C for 20 min.

*Y* = 8.59 × 10^7^*Z*^1.3627^ (1)

where *Y* is the cell concentration (cells/mL), and *Z* is the OD_600_ value.

### 2.3. Specimen Preparation

#### 2.3.1. Test Device

The sample mold was an acrylic tube with an outer diameter of 45 mm, a wall thickness of 3 mm, an inner diameter of 39 mm, and a height of 100 mm, cut into two semicircular C-shaped acrylic tubes. First, the upper and lower ends of the pipe were fixed with pipe hoops, the bottom end of the pipe was wrapped with 450-mesh nylon cloth, and gravel with a particle size of about 2 mm was added to the pipe at a height of about 10 mm, and 60-mesh nylon cloth was laid on the upper end of the gravel. Then, the dried and pulverized pyrite tailings were added, 60-mesh nylon cloth was laid on the upper end of the pyrite tailings, and gravel with a particle size of about 2 mm was added to the top of the pipe. The dry density of the samples was 1.60 g/cm^3^, which was the optimum dry density of MICP-biocemented fine sand.

#### 2.3.2. Curing Test

In order to explore the effect of grouting times on the curing effect under optimal conditions [32]—i.e., an OD_600_ value of the bacterial liquid of 1.8, a concentration of cementitious liquid of 1.0 mol/L, and a urea-to-calcium-chloride ratio of 1:1—the samples were grouted in different rounds. The grouting round was set to 1, 2, 3, 4, 5, and 6 rounds, and each sample was injected with a bacterial solution once and a cementation solution twice in each round. The injection rate of the bacterial solution and cementation solution was 0.9 mL/min, which was automatically controlled by a peristaltic pump. Each injection volume was 30 mL, which was half of the pore volume of the sample. The liquid was fully contacted and then injected a second time. After 1 to 6 rounds of grouting, the samples were placed in a curing box and cured for 45 days. Then, they were dried and demolded at 70 °C for subsequent tests.

For the convenience of recording and subsequent experimental data processing, each sample was numbered as A, B, C, D, E, or F. The untreated tailings were set as the control group. Three parallel samples were used for the UCS test, two parallel samples were used for the permeability test, and the other sample was reserved for standby.

### 2.4. Tests

#### 2.4.1. UCS

In accordance with the standard test method for UCS of the immobilized mine tailings (ASTM D2166/D2166M-16 2016), the as-prepared specimen was assembled in a strain-controlled YHS-2 UCS testing apparatus. The UCS test was then performed at a vertical strain rate of 1 %/min. The stress and strain of the specimen were recorded at intervals of 5 s until the specimen failed. Three parallel samples of each group were used to test UCS, and the average value was taken according to the test results.

#### 2.4.2. Permeability Test

According to the specification “Highway Geotechnical Test Regulations” (JTGE40-2007), a permeability test was carried out on the samples that completed the specified number of grouting tests. The test used a flexible wall permeameter to conduct a constant head permeation test on the partially cured samples. The volume of water injected at the backpressure end was verified during the permeation test, and the corresponding time was recorded. Finally, the permeability coefficient was calculated according to Darcy’s law. Two parallel samples of each group were used to test the permeability, and the average value was taken according to the test results.

#### 2.4.3. Pollution Components

The curing effect of the contaminated soil was evaluated using the toxic leaching test method (TCLP) recommended by the Resource Conservation and Recovery Act (RCAC) in the United States [35]. The unconfined compressive strength test samples were collected, crushed, and dried for the TCLP test, and the leachate obtained was filtered using a 0.22 μm filter membrane. A part of the collected filtrate was subjected to an inductively coupled plasma mass spectrometry test (ICP–MS) using a 7500 Series instrument produced by Agilent Technologies (California, CA, USA). Another part of the filtrate was subjected to ion chromatography detection. Finally, the filtrate was collected after each grouting round and its pH was detected.

#### 2.4.4. Micromorphology Testing

One sample was selected from each group of samples after curing: A, B, C, D, E, and F, plus the control group. A total of seven samples were tested; the samples were placed in an oven for drying, crushed, passed through a 200-mesh sieve, and used for XRD (Hefei, China), TGA (Hefei, China), and SEM (Hefei, China) to analyze the chemical bonds and chemical components in the cured samples. 

XRD patterns were obtained using a Rigaku D/Max-2005 V diffractometer with Cu-Kα scanning from 5 to 80° 2θ. The components in the samples were identified by comparison with standards established by the International Center for Diffraction Data. 

The instrument used in the TGA test was a STA449F3 synchronous thermal analyzer manufactured by NETZSCH, Germany. During the test, the atmosphere was nitrogen (flow rate 50 mL/min), the temperature range was set from 25 °C to 1000 °C, and the heating rate was 5 °C/min. The FTIR test used a Nicolet iS50 FTIR spectrometer manufactured by Thermo Fisher Scientific. The sample was prepared via the tableting method during the test, and the measurement range was 4000~400 cm^−1^.

SEM imagery was obtained to provide direct evidence of reaction product analyses. Samples of approximately 5 × 5 × 5 mm were selected; they were initially freeze-dried before their surfaces were cleaned and then coated with gold, in preparation for SEM analysis.

## 3. Results and Discussion

### 3.1. Effect of Curing Rounds on Engineering Properties of Tailings 

#### 3.1.1. Unconfined Compressive Strength

**Figure 2:**
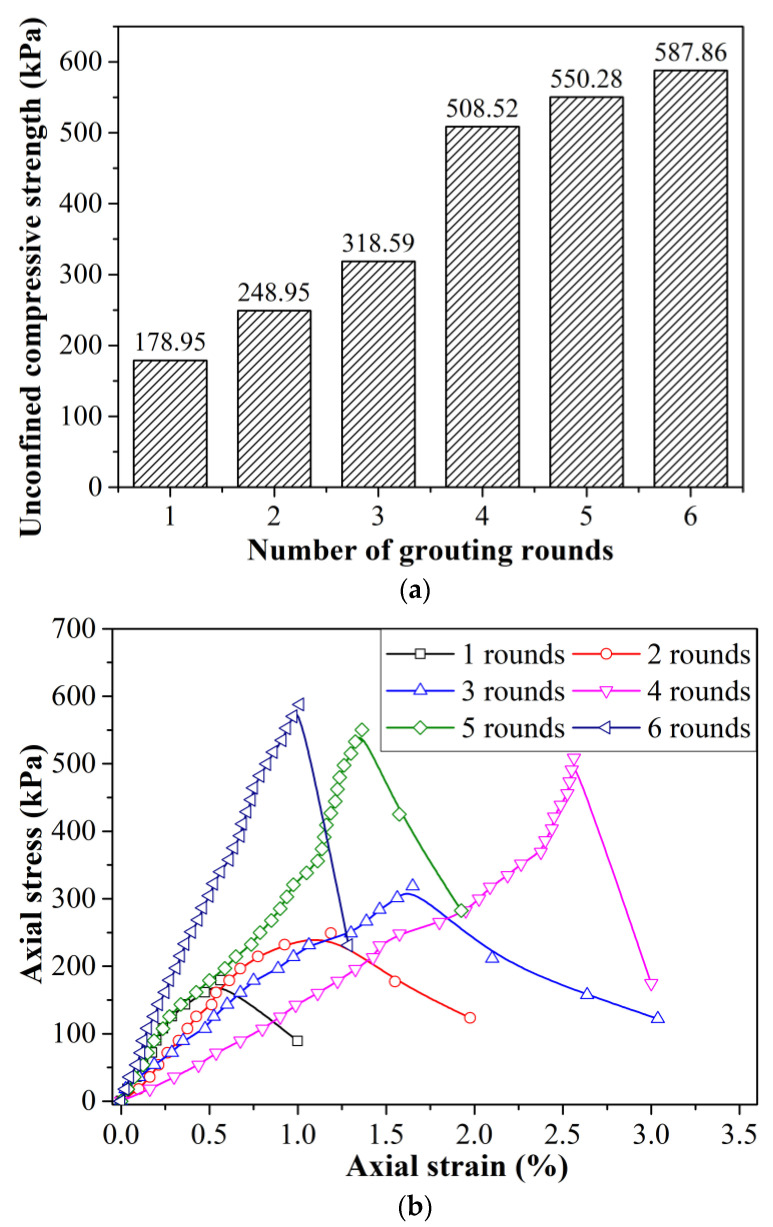
Strength characteristic curve of biocemented tailings with different grouting rounds: (**a**) UCS diagram of biocemented tailings with different grouting rounds; (**b**) stress–strain curve of biocemented tailings with different grouting rounds.

The unconfined compressive strength test data of the biocemented pyrite tailings are shown in Figure 2. As can be seen from the figure, with an increase in the number of grouting rounds, the unconfined compressive strength of the sample showed an upward trend. However, the overall unconfined compressive strength was not very high, reaching 500 kPa in 4–6 rounds of grouting. The highest value was recorded for the six-round grouting sample, which reached 587.86 kPa. However, comparing the results of the fifth and sixth rounds, the strength did not increase much, and the sample was difficult to infiltrate during the sixth round of grouting; at this point, most of the pores in the sample were filled, which also shows that a larger number of grouting rounds is not necessarily better.

With an increase in grouting rounds, the strength of the sample continued to increase, the stress–strain relationship of the sample gradually transformed from a strain-hardening type to a strain-softening type, and the failure mode gradually transitioned from a plastic failure mode to a brittle failure mode. After four rounds of grouting, the failure mode of the sample changed to the strain-softening type, and the failure mode was a brittle failure.

**Figure 3:**
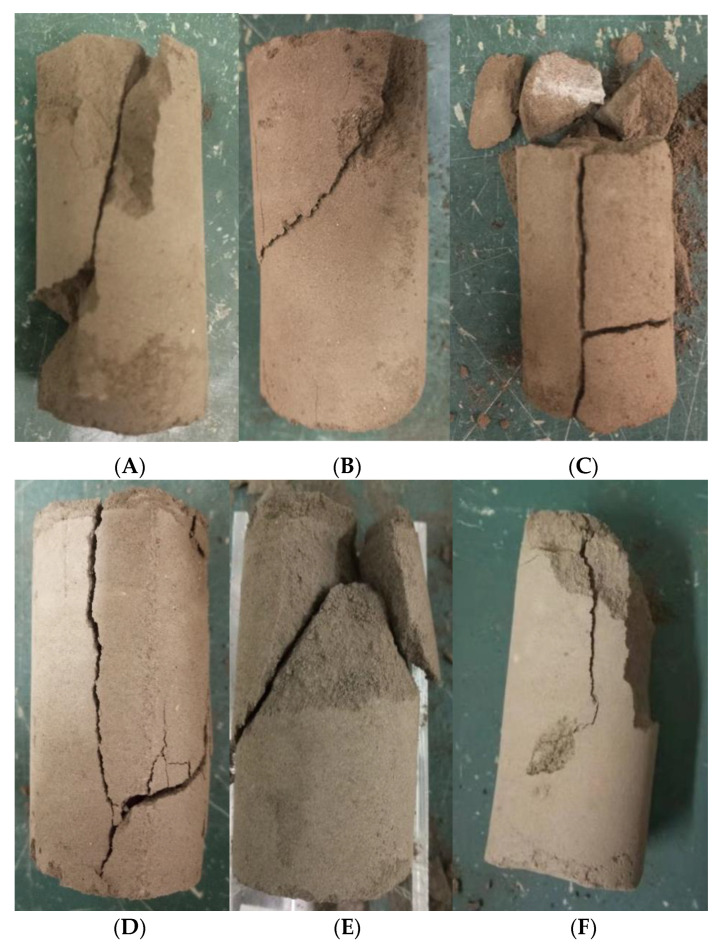
(**A**–**F**)The failure diagrams of samples with 1–6 rounds of grouting.

The failure states of the samples with one to six rounds of grouting are shown in Figure 3. It can be seen from Figure 3 that the samples of grouting rounds 1 and 2 (A and B, respectively) showed shear failure from the top at 45°, and the samples of grouting rounds 3 and 4 (C and D, respectively) all exhibited splitting through the sample. The samples with five and six rounds of grouting (E and F, respectively) had similar failure modes to the samples with one or two rounds of grouting (A and B, respectively).

#### 3.1.2. Permeability Test

**Figure 4:**
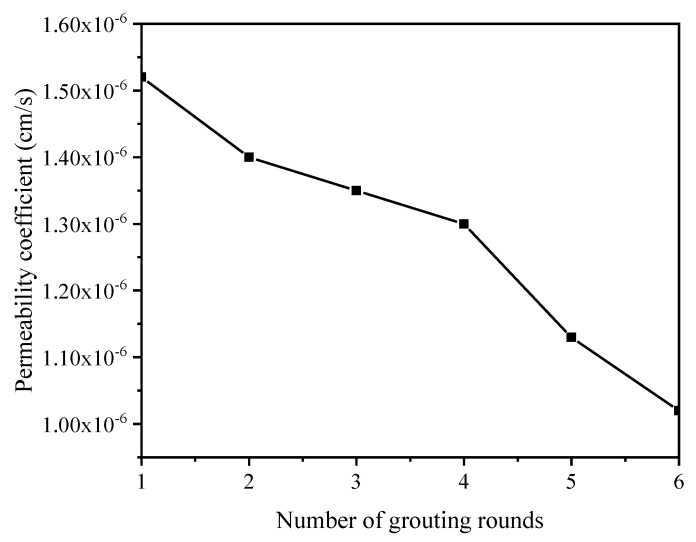
Permeability coefficient curve of samples with 1–6 rounds of grouting.

The permeability coefficient results are shown in Figure 4. It can be seen from the figure that, with an increase in the number of grouting rounds, the permeability coefficient of the sample gradually decreased. The permeability coefficient of sample E reached 1.13 × 10^−6^ cm/s. The initial permeability coefficient of the old tailing sand in the Xinqiao mining area was 5.10 × 10^−3^ cm/s, and the permeability coefficient of the new tailing sand was 2.79 × 10^−3^ cm/s [36]. The tailings sand used in this test was the new tailings sand of Xinqiao pyrite. MICP biocementation reduced the permeability coefficient of the tailings by three orders of magnitude and reached the magnitude of clay soil, which showed that the MICP-cured pyrite tailings sand had a good effect. These results indicate that MICP biocementation technology can effectively reduce the permeability of tailings and form a layer on the surface of tailings to inhibit their oxidation. This parameter indicated that most of the pores in the sample were filled, which could effectively prevent the migration of heavy metal elements and the oxidative release of S elements. 

### 3.2. Influence of Curing Rounds on the Environmental Effects of Tailings Sand

#### 3.2.1. pH

The results for the pH value of each filtrate group are shown in Table 3. From the experimental results, with an increase in the number of grouting rounds, the overall trend of the pH of the filtrate showed an upward movement. However, the sample was still slightly acidic and gradually changed to neutrality under MICP curing, which also showed that the MICP technology was effective for S elements and had a good curing effect. 

#### 3.2.2. Sulfur Element

**Figure 5:**
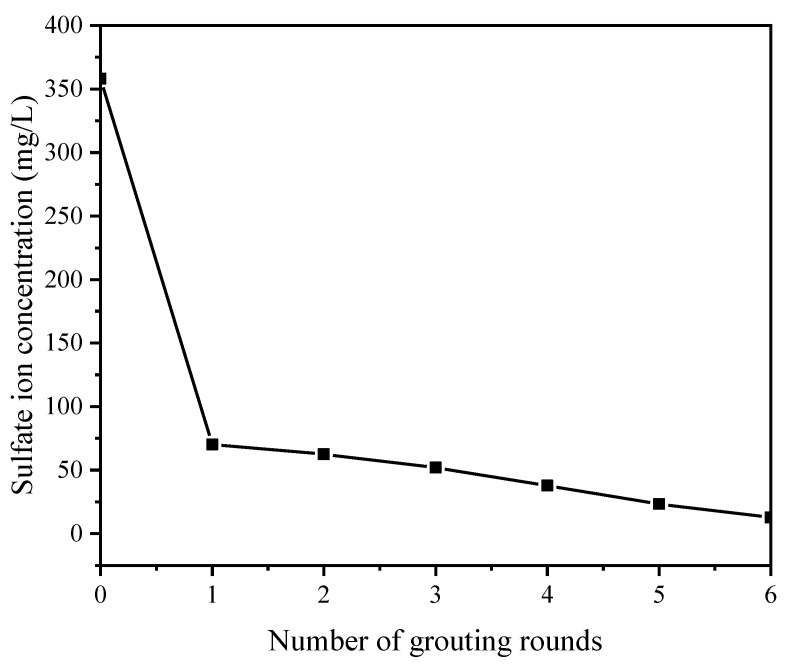
SO_4_^2−^ content variation in samples with 0–6 rounds of (0 represents the control group).

It can be seen from Figure 5 that the SO_4_^2−^ content of the experimental group was significantly lower than that of the control group, and with an increase in the number of grouting rounds, the SO_4_^2−^ content became lower and lower, and the SO_4_^2−^ content dropped to 70.16 mg/L after one round of grouting. The SO_4_^2−^ content was 12.88 mg/L after six rounds of grouting. 

#### 3.2.3. Heavy Metals

For the convenience of comparison, this paper introduces the concept of a “removal rate” to more intuitively reflect the effect of microorganisms that induce calcium carbonate precipitation to biocement pyrite tailings sand. The removal rate of heavy metals can be calculated by Equation (2).

(2) $$Removal\;rate=1-\frac{c_{m}}{c_{0}}$$

where *C_m_* is the leaching concentration of heavy metals after biocementation, and *C*_0_ is the leaching concentration of heavy metals in the uncemented sample. The removal rate is expressed as a percentage.

**Figure 6:**
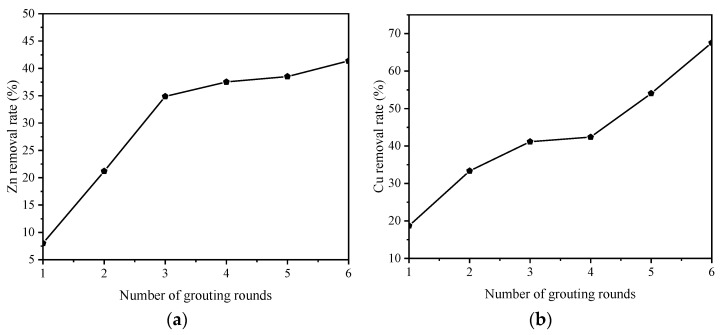

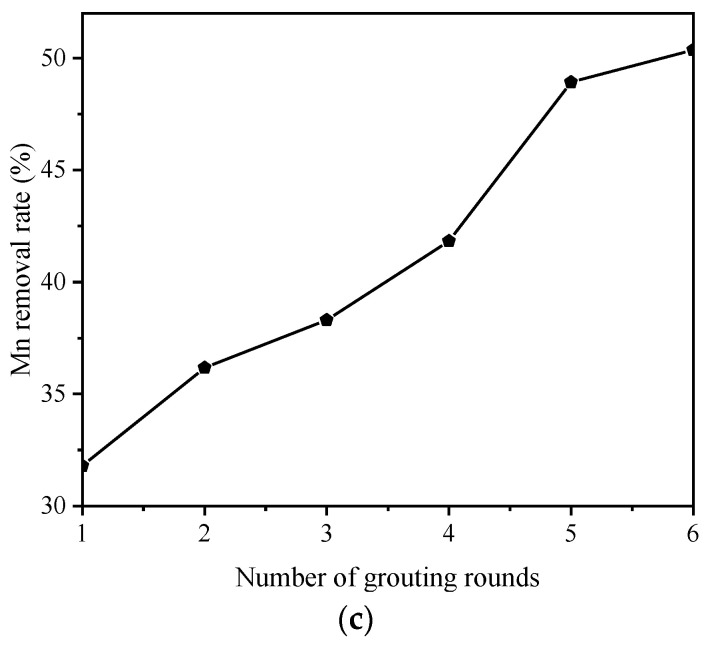
Changes in the removal rate of common heavy metals from samples with 1–6 rounds of grouting: (**a**) result for Zn; (**b**) result for Cu; (**c**) result for Mn.

As shown in Figure 6, with an increase in the number of grouting rounds, the content of several heavy metals in the filtrate showed a downward trend.

Overall, the removal rate of Cu was the highest, followed by the removal rate of Mn; the removal rate of Zn was the lowest. However, it was also close to 50%, indicating that *Bacillus pasteurii* has a robust biocementation effect on heavy metals. The contents of heavy metal elements in the biocemented samples were all lower than the above standards (GB 36600-2018, GB 15618-2018).

### 3.3. Microscopic Test Results

#### 3.3.1. XRD Results 

**Figure 7:**
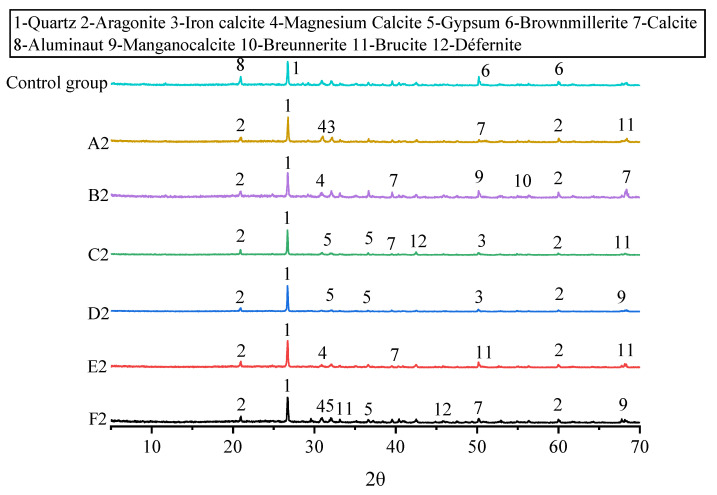
XRD results of samples with 0–6 rounds of grouting.

The XRD analysis and detection results are shown in Figure 7. The position and number of wave peaks in the control samples and the biocemented sample were different. The results of A~F and the control group were analyzed, and new minerals were found that had been biocemented by MICP in the pyrite tailings sand.

In the analysis, bauxite, quartz, and hemoranite were the main minerals found in the control group. Aragonite, iron calcite, magnesium calcite, gypsum, calcite, manganese-rich calcite, iron-rich magnesite, brucite and were found in the MICP-biocemented the pyrite tailings sand. Most of the newly formed minerals existed in metal carbonates form. A small number of minerals were found in the form of metal sulfates and hydroxides, which indicates that metal cations are combined with carbonate ions to achieve the effect. 

#### 3.3.2. FTIR Results 

**Figure 8:**
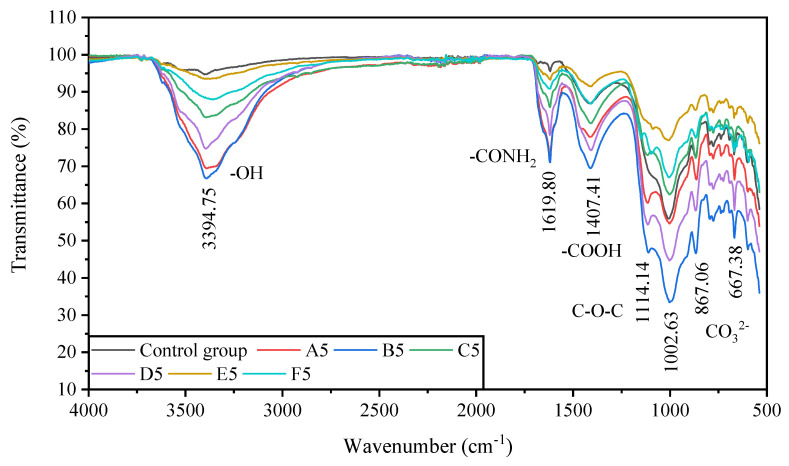
The FTIR results of samples with 0-6 rounds of grouting.

The FTIR analysis and detection results are shown in Figure 8. The vibrational peak bands of each spectrum included hydroxyl absorption bands at 2750~3750 cm^−1^, oxygen-containing functional groups at 900~1750 cm^−1^, and mineral absorption bands at 650~800 cm^−1^; the peaks appeared at 3394.75 cm^−1^, 1619.80 cm^−1^, 1407.41 cm^−1^, 1114.14 cm^−1^, 1002.63 cm^−1^, 867.06 cm^−1^, and 667.38 cm^−1^. The main oxygen-containing functional groups were carboxyl-COOH, hydroxyl-OH, amide-CONH_2_, and ether bond C-O-C, indicating that organic and inorganic reactions occurred simultaneously during the biocementation of pyrite tailings sand by MICP. 

#### 3.3.3. TGA Results 

**Figure 9:**
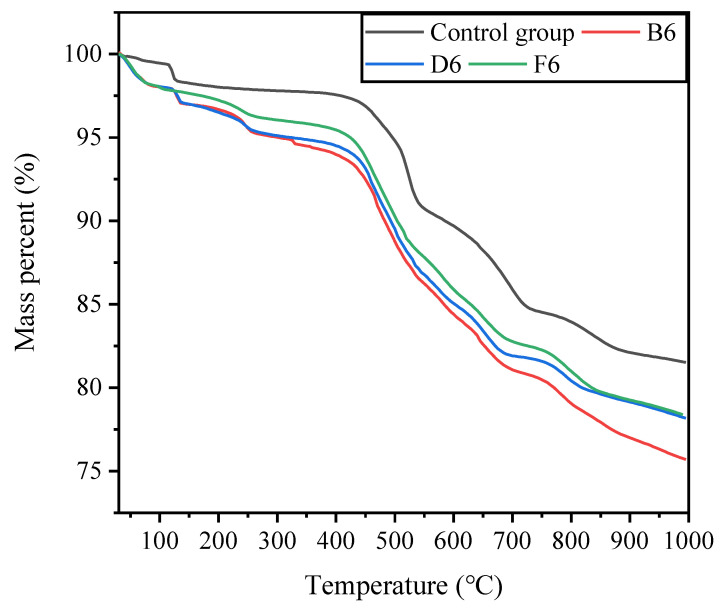
The results of TG.

**Figure 10:**
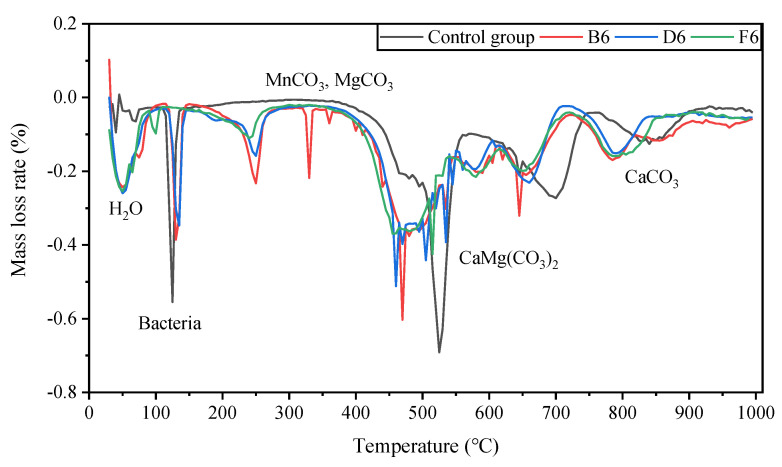
DTG results of classic samples.

When a sample is thermally decomposed, its quality will change, and TG and DTG analysis can reflect the thermal stability and pyrolysis of the sample. Figure 9 and Figure 10 show the TG and DTG curves of the test samples, respectively. Overall, the pyrolysis process of the cured samples showed a similar trend and had six stages. The first stage was from the initial temperature to 80 °C and was labeled as the water evaporation stage. As the temperature increased, the water evaporation speed increased, reaching a maximum value of about 50 °C, which is the decomposition of bacterial and extracellular polymeric substances (EPSs). The range of 200 °C~1000 °C was the decomposition stage of the mineral components in the sample.

This section will mainly analyze key minerals according to the results of the XRD analysis. At 300 °C~550 °C, the main processes were the decomposition of MnCO_3_, MgCO_3_, and gypsum; at 550 °C~750 °C, the main process was CaMg(CO_3_)_2_ in the decomposition of Mg element; at 800 °C~1000 °C, the main process was the decomposition of CaCO_3_, which can confirm the mechanism of action of MICP for the solidification of pyrite tailings sand.

#### 3.3.4. SEM Results

**Figure 11:**
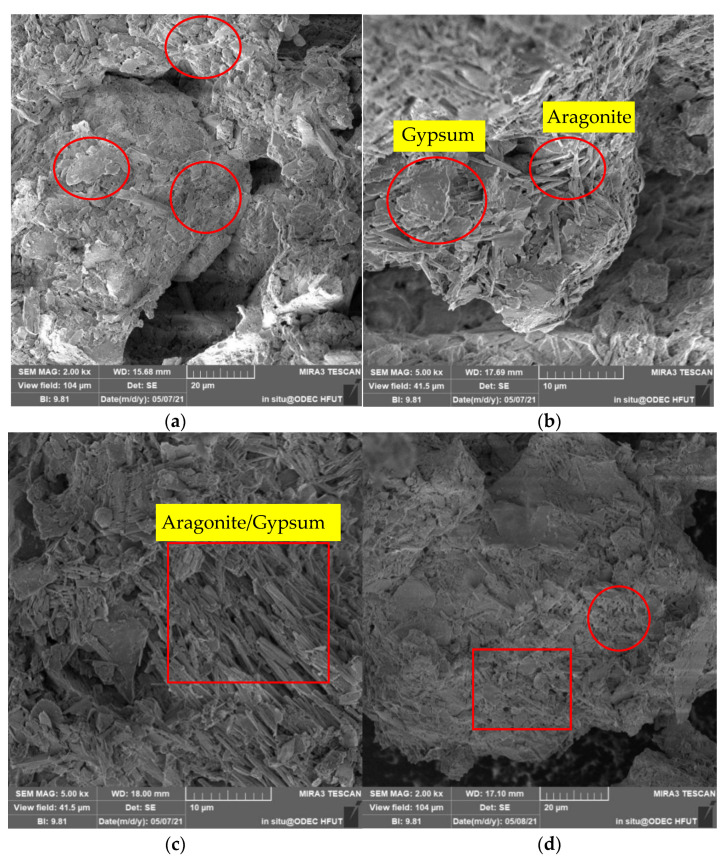

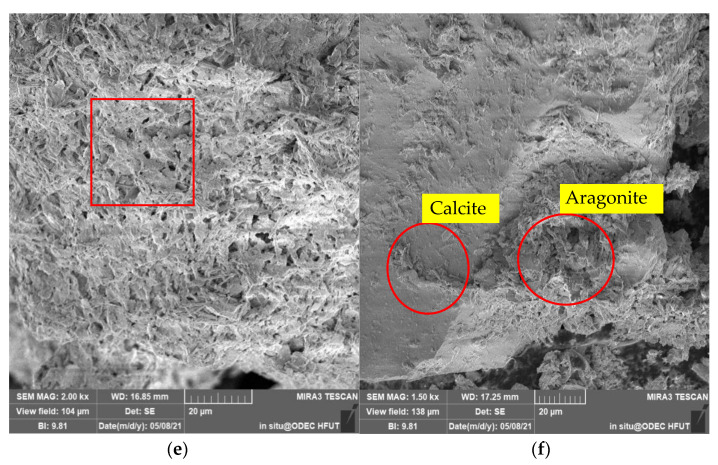
SEM results for classic samples after 0–6 rounds of grouting: (**a**) control group 2000X magnification; (**b**) B1 5000× magnification; (**c**) C1 5000× magnification; (**d**) E1 2000× magnification; (**e**) E1 2000× magnification; (**f**) F1 1500× magnification.

The SEM results are shown in Figure 11. Figure 11a shows an image of a sample from the blank control group magnified 2000 times. It can be seen from the figure that the un-treated pyrite tailings had many voids and were very loose. In Figure 11b, an image of the B1 sample magnified 5000 times clearly shows columnar minerals and tabular minerals, which were judged to be gypsum and aragonite according to their size. In the image of the C1 sample magnified 5000 times, it can also be seen that there were a large number of acicular minerals and a small number of spherical minerals. Combined with the XRD results, it was judged that these were aragonite or gypsum. Figure 11d,e both show images of the E1 sample magnified 2000 times. It can be seen that short columnar and flake minerals were cemented together, and there were some small pores during this period, indicating that MICP has a good cementation effect. From Figure 11f, it can be seen that, after XRD treatment, the surface of the sample was in the shape of short calcite and aragonite, and the bonding effect was obvious.

### 3.4. The Mechanism for the Biocementation of Pyrite Tailings Sand by MICP

**Figure 12:**
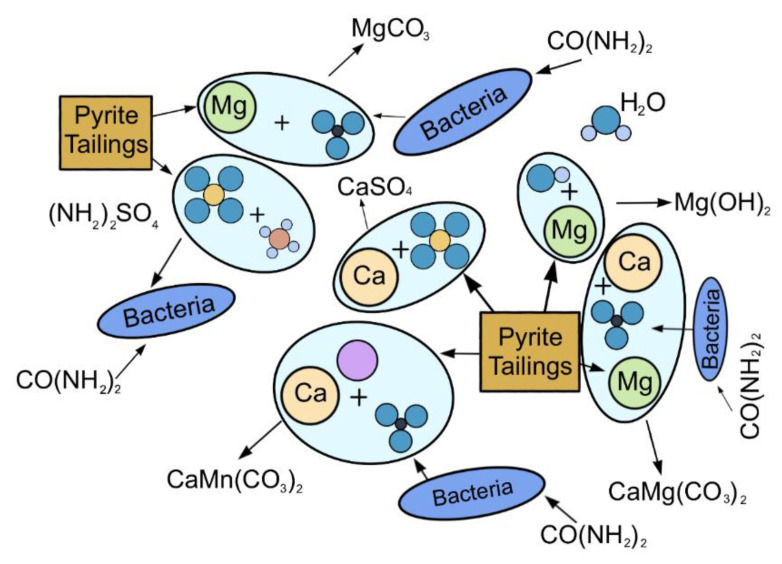
Schematic diagram of the mechanism of MICP solidified pyrite tailings.

A comprehensive analysis of the XRD, SEM, FTIR, and TGA findings and the results of the previous sections showed the presence of heavy metals and sulfur elements in the MICP-cemented pyrite tailings mainly through co-precipitation and biological action. Here, abiotic and biological mechanisms are introduced to explain MICP-immobilized pyrite tailings [37]. The action mechanism of MICP-cemented pyrite tailings is shown in Figure 12.

#### 3.4.1. Abiotic Mechanisms

**Figure 13:**
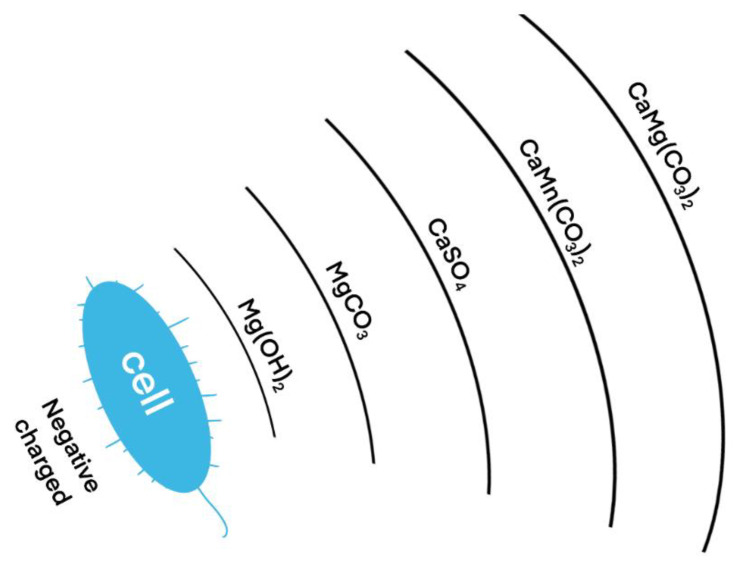
Hypothetical diagram of multilayer precipitation structure.

The non-biological mechanism refers to the reactions between heavy metals and substances contained in the grouting fluid to form precipitates. The bacterial solution was injected into the sample first during the grouting process, and then the cementing solution was injected. The pH value of the filtrate gradually increased, which was beneficial to the acid–base balance. After the cementing solution is added, urea is hydrolyzed to generate CO_3_^2−^ and NH_4_^+^. At this time, the heavy metal ions in the sample and Ca^2+^ in the cementing solution will combine with CO_3_^2−^ to form metal carbonate and calcium carbonate. In the XRD results, aragonite, iron calcite, magnesium calcite, gypsum, calcite, manganese-rich calcite, iron-rich magnesite, brucite, and berberlite were found, the main components of which are CaCO_3_, CaMg(CO_3_)_2_. CaSO_4_, CaMn(CO_3_)_2_, MgCO_3_ and Mg(OH)_2_. Due to the presence of other heavy metal elements in the pyrite tailings, it was difficult to deduce the sequence of precipitation formation through the test process. However, according to the existing results, with an increase in the number of grouting rounds, the main sediments appeared as follows: Mg(OH)_2_ → MgCO_3_ → CaSO_4_ → CaMn(CO_3_)_2_ → CaMg(CO_3_)_2_. From this, a hypothesis of a multilayer precipitation structure was developed, as shown in Figure 13.

#### 3.4.2. Biological Mechanism

The biological mechanism refers to the consumption or precipitation of heavy metal ions through biological actions, which mainly include biosorption and bioaccumulation. Biosorption refers to the capture of heavy metal ions through the cell wall of microorganisms and the subsequent adsorption of the ions to the binding sites on the cell wall [38]. Bioaccumulation refers to the entry of heavy metal ions into the cytoplasm through the cell membrane during cell metabolism.

The FTIR results showed that the main oxygen-containing functional groups in the cured samples were carboxyl-COOH, hydroxyl-OH, amide-CONH_2_, and ether bond C-O-C. These groups proved the role of bacteria in this process. The liquid medium contained yeast extract, which is a required nutrient for bacterial growth. Yeast extract contains a large number of proteins, and each protein contains at least one carboxyl-COOH and one amino-NH_2_; The appearance of amide-CO-NH_2_- and ether bond C-O-C confirmed the bacterial metabolism [39]. In addition, according to the recommended medium containing (NH_4_)_2_SO_4_ for *Bacillus Pasteurella* (US National Culture Bank No. ATCC11859), it can be seen that urea is decomposed by *Bacillus Pasteurella* to generate CO_3_^2−^ and NH_4_^+^. In contrast, the sulfur element in pyrite is decomposed by *Bacillus Pasteurella*. Oxidation generates SO_4_^2−^, and the SO_4_^2−^ in the sample will generate (NH_4_)_2_SO_4_ when it encounters NH_4_^+^, thereby providing the substances needed for the growth of bacteria, which is also one of the reasons for the decrease in the content of SO_4_^2−^ in the sample.

As *Bacillus pasteurii* is negatively charged, it can attract positively charged metal cations. Through the metabolic activity of *Bacillus pasteurii*, heavy metal ions are wrapped outside the bacteria, forming the multilayer structure described above. As can be seen from our results, the innermost layer is Mg(OH)_2_, which is converted into MgCO_3_ in the second layer with the passage of time; the third layer consists of CaSO_4_, SO_4_^2−^ released from pyrite tailings sand, and cementitious liquid. A combination of Ca^2+^ covers the outer layer, which can prevent the valence state transition of Mg^2+^ to a certain extent; the fourth and fifth layers are CaMn(CO_3_)_2_ and CaMg(CO_3_)_2_, indicating that Mn^2+^ and Mg^2+^ are gradually transformed into stable compounds. It can be speculated that over time, Mn^2+^ will gradually transform into a more stable compound form.

## 4. Conclusions

Through the introduction of the research background, test methods, engineering properties, environmental effects of cured samples, and microscopic analysis, the following conclusions were obtained:

(1) It is feasible to use MICP technology to biocement pyrite tailings sand. The UCS increased significantly and the permeability coefficient decreased to that of clay. In the TCLP test of the cured samples, the ion concentrations of Mn^2+^, Zn^2+^, and Cu^2+^ and the ratio of the control group all fell below the standard. The content of SO_4_^2−^ was significantly reduced.
(2) The microscopic analysis showed that MICP biocemented pyrite tailings mainly produce various carbonate minerals (e.g., aragonite, iron calcite, magnesium calcite, calcite, manganese-rich calcite, iron-rich magnesite, brucite, and carbortite) and gypsum. The FTIR results showed that CO_3_^2−^ is generated. The TGA results corroborated the XRD results.
(3) The action mechanism of the microorganism-induced calcium carbonate precipitation and biocementation of pyrite tailings sand mainly includes the following: urea hydrolysis, microbial utilization after sulfur oxidation, and heavy metal ion fixation. In these processes, complex biochemical and physicochemical reactions occur, which finally induce calcium carbonate precipitation to achieve the biocementation of heavy metals and sulfur elements.
(4) MICP biocementation technology can effectively reduce the permeability of tailings and form a layer on the surface of tailings by carbonate precipitation to inhibit their oxidation. This results in a reduction in the concentration of heavy metals and controls their mobility through microbial adsorption, intracellular accumulation, and coprecipitation.

## Figures and Tables

**Table 1:** Sample tailings XRF analysis and datasheet.

Component	Quality Score (%)	Contains Elements	Elemental Content per Kilogram (mg)
SiO_2_	30.61	Si	14,284,666.67
Fe_2_O_3_	21.52	Fe	230,554.52
SO_3_	19.52	S	168,028.16
CaO	10.87	Ca	151,558.86
Al_2_O_3_	7.62	Al	21,925.43
K_2_O	2.10	K	6639.13
MgO	1.42	Mg	1789.20
MnO	1.08	Mn	1188.00
ZnO	0.07	Zn	60.67
CuO	0.06	Cu	3.35
PbO	0.03	Pb	1.67

**Table 2:** Contents of heavy metal elements and SO_4_^2–^ in sample tailings.

Leached Iron	Cu^2+^	Zn^2+^	Mn^2+^	Pb^2+^	SO_4_^2−^
**Content (mg/kg)**	0.50	0.45	27.18	0.03	358.21

**Table 3:** pH changes in samples during 1–6 rounds of grouting.

Sample	pH					
A	5.91	-	-	-	-	-
B	6.09	6.22	-	-	-	-
C	5.96	6.23	6.37	-	-	-
D	6.03	6.16	6.21	6.30	-	-
E	5.94	6.15	6.32	6.47	6.60	-
F	5.98	6.18	6.36	6.37	6.44	6.69

## Data Availability

Not applicable.

## Sample Availability

Samples of the compounds are not available from the authors.
